# Ever-evolving: introducing the Medical Heritage Library, Inc

**DOI:** 10.5195/jmla.2019.651

**Published:** 2019-04-01

**Authors:** Emily R. Novak Gustainis

**Affiliations:** Vice-President, Medical Heritage Library, Inc., and Deputy Director, Center for the History of Medicine, Francis A. Countway Library of Medicine, Harvard University, Cambridge, MA, emily_gustainis@hms.harvard.edu

## Abstract

The Medical Heritage Library, Inc. (MHL), is a collaborative digitization and discovery organization committed to providing open access to history of medicine and health resources. Since its founding in 2010, it has aspired to be a visible, research-driven history of medicine and health community that serves a broad, interdisciplinary constituency. The MHL’s goal is to make important historical medical content, derived from leading medical libraries, available online free of charge and to simplify and centralize the discovery of these resources. To do so, it has evolved from a digitization collaborative of like-minded history of medicine libraries, special collections, and archives to an incorporated entity seeking not just to provide online access to digital surrogates, but also to embrace the challenges of open access, the retention and use of records containing health information about individuals, and service to the digital humanities. This organizational expansion was further spurred by the MHL’s recently completed National Endowment for the Humanities grant, “Medicine at Ground Level: State Medical Societies, State Medical Journals, and the Development of American Medicine” (PW-228226-15), which received additional financial support from Harvard Medical School and the Arcadia Fund through the Harvard University Library.

## FROM THE MEDICAL HERITAGE LIBRARY TO MEDICAL HERITAGE LIBRARY, INC

The Medical Heritage Library, Inc. (MHL), facilitates much needed discourse about the contemporary practice of medicine and surgery, dentistry, mental health, nursing, public health, veterinary medicine, and the biological sciences, as well as associated subjects ranging from art and gardening to physical culture and alternative medicine.

While the majority of texts are in English, the MHL includes works in French, Spanish, German, Latin, Portuguese, and Dutch. Materials are selected for their scholarly, educational, and historical value and include monographs, pamphlets, ephemera, and video dating from circa 1200 to the 2000s. Many of the works are richly illustrated and address cultural and temporal perceptions of the body; race, gender, and ethnicity; the mechanics of medicine; and the rise of new industries built to serve or contest status-quo medical practices. MHL content is freely available via the Internet Archive, with metadata for all collection items searchable in the Digital Public Library of America (DPLA). Examples of images from MHL texts accompany this article.

As of December 12, 2018, the MHL stood at 273,548 items, with a total of 56,017,982 page views and 73,599,986 downloads since June 1, 2010. The geographic reach of the MHL is global. Page views within 30 days of authoring this article were dominated by users in Virginia, California, New York, Oregon, and Texas (in that order); the Ile de France region of France and regions in Germany; the Netherlands; and the United Kingdom.

**Figure:**
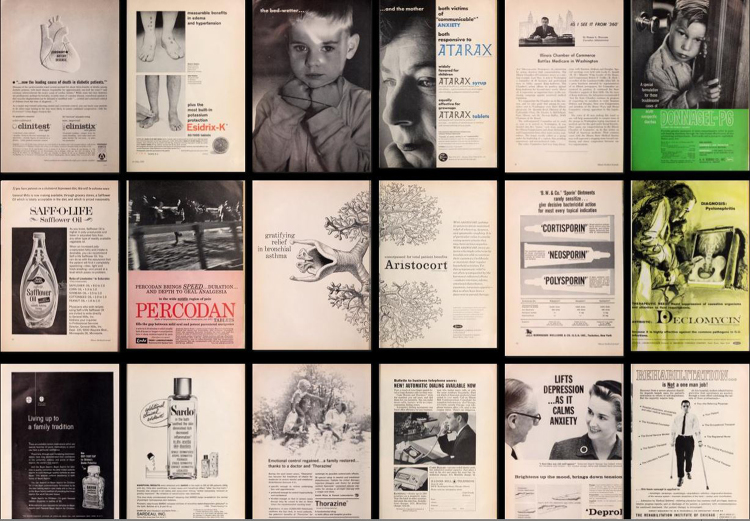
Advertisements from the July 1962 issue of the *Illinois Medical Journal*, the journal of the Illinois State Medical Society.

**Figure:**
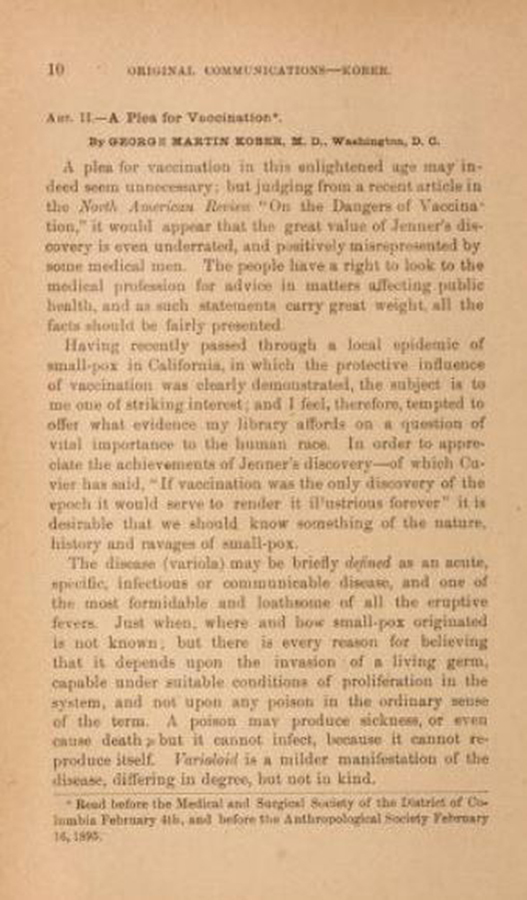
First page of the article, “A Plea for Vaccination” by George Martin Kober, MD, in the April 1895 issue of the *Virginia Medical Monthly*, the journal of the Medical Society of Virginia.

MHL content has been primarily built through four successive grants: *“*Medical Heritage Library Phase I: 2010–2011” (Alfred P. Sloan Foundation); “Planning for an Innovative Partnership: The Medical Heritage Digital Collaborative*: 2*011–2012” (National Endowment for the Humanities Digital Start-Up Grant); “Expanding the Medical Heritage Library: Preserving and Providing Online Access to Historical Medical Periodicals: 2012–2014” (National Endowment for the Humanities, Humanities Collections and Reference Resources); and “Medicine at Ground Level: State Medical Societies, State Medical Journals, and the Development of American Medicine and Society: 2014–2017” (National Endowment for the Humanities).

Between 2013 and 2014, the MHL also administered the Council on Library and Information Resources Hidden Collections Program grant, “Private Practices, Public Health: Privacy-Aware Processing to Maximize Access to Health Collections” (Andrew W. Mellon Foundation). This grant opened nine manuscript collections of public health researchers and health policy makers vested in the economic, ethical, legal, and social aspects of health care; HIV and nutrition research; and community mental health. While not a digitization grant, this work fostered a dialogue between historians and archivists about approaches to facilitating access to collections containing health information about individuals in Health Insurance Portability and Accountability Act (HIPAA) and non-HIPAA covered entities.

The MHL incorporated in May 2018. It now consists of nine principal members with governance and voting rights and more than thirty nonvoting contributors with MHL-tagged content in the Internet Archive. The MHL’s membership structure consists of two types of principals: dues-paying and providers of much needed in-kind service to support MHL administration, communications, and discovery efforts. Current principal members include the Augustus C. Long Health Sciences Library at Columbia University, Bibliothèque interuniversitaire de santé (BIU Santé), the College of Physicians of Philadelphia, Cushing/Whitney Medical Library at Yale University, Francis A. Countway Library of Medicine at Harvard University, the New York Academy of Medicine, University of California–San Francisco Library and Center for Knowledge Management, the National Library of Medicine at the National Institutes of Health, and the Wellcome Library and UK-MHL partners. The MHL is always seeking new principal members and content contributors.

## NEW CHALLENGES AND OPPORTUNITIES: STATE MEDICAL SOCIETY JOURNALS AND “Medicine at Ground Level”

The rationale for the MHL, and all of its subsequent activities, is best articulated in its 2009 appeal to the Alfred P. Sloan Foundation, whose funding launched the MHL:

Perhaps no subject has been so fundamental to understanding and interpreting the human condition as has medicine. Such interpretations entail concerns regarding birth and death, health and disease, and the normal and the pathological. Around such concerns, systems of knowledge, patterns of care, and professions and para-professions have been developed in various contexts, conditioned by related belief systems and political economic configurations…Given the centrality of such concerns to the human condition, the “history of medicine” itself often transcends such disciplinary boundaries, extending to the interests of general and economic historians, classicists and archaeologists, historians of religion and art, linguists and literary scholars, philosophers and historians of ideas, and sociologists and policy-makers.

The relationship between “medical” resources and these more diverse audiences helped to inspire our “Medicine at Ground Level” project. The state medical society journals document the transformation of American medicine at both the local and national level, and serve as sites not only for scientific articles, but also for medical talks, local news regarding the medical profession, pharmaceutical and device advertising, and uncensored musings on medicine and society throughout the twentieth century. Yet the broader audiences for such a project were articulated by former president of the American Association for the History of Medicine, Distinguished Professor of History Nancy J. Tomes, Stony Brook University, who noted in her letter of support:

The value of this collection lies precisely in the insights state journals provide on issues of great contemporary interest. They shed light on questions at the heart of today’s policy debates: why do physicians treat specific diseases so differently in different parts of the country? Why is it such a challenge to develop and implement professional policies at the national level? How do state level developments in health insurance influence federal policy and vice versa? How do factors such as race, class, gender, and ethnicity affect therapeutic decision making? How have methods of promoting new therapies and technologies changed over time? These are issues of interest not only to historians but to political scientists, sociologists, and economists.

Over the past year, the MHL has provided researchers with free online resources to examine opiate addiction in the aftermath of Civil War, the impact of the 1918 Spanish influenza epidemic, the history of sedatives, and the varied uses of the term “personal equation” in medical literature from the 1850s to the 1950s. The MHL has also fostered digital humanities investigations like identifying parallel passages across disciplines using MHL-digitized texts.

**Figure:**
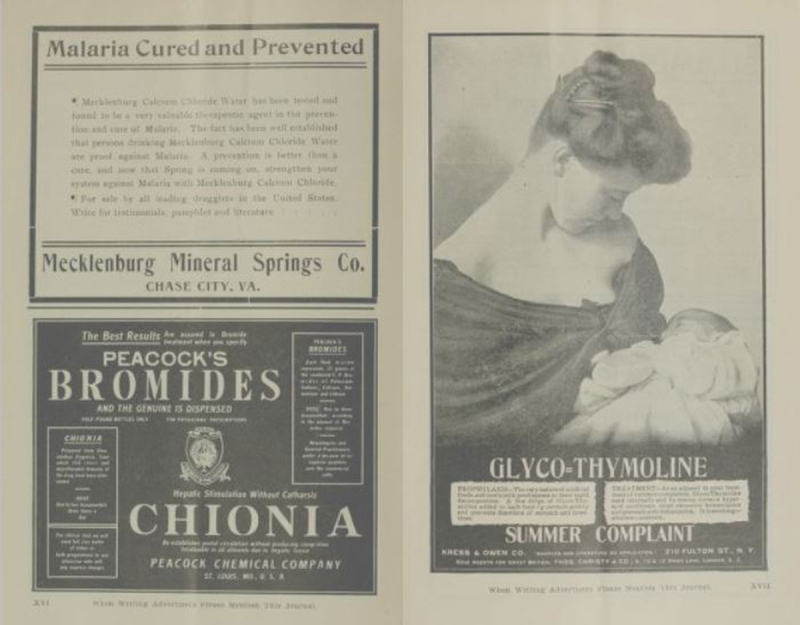
Advertising spread from the July 1905 issue of the *Carolina Medical Journal*, the journal of the North Carolina Medical Society.

**Figure:**
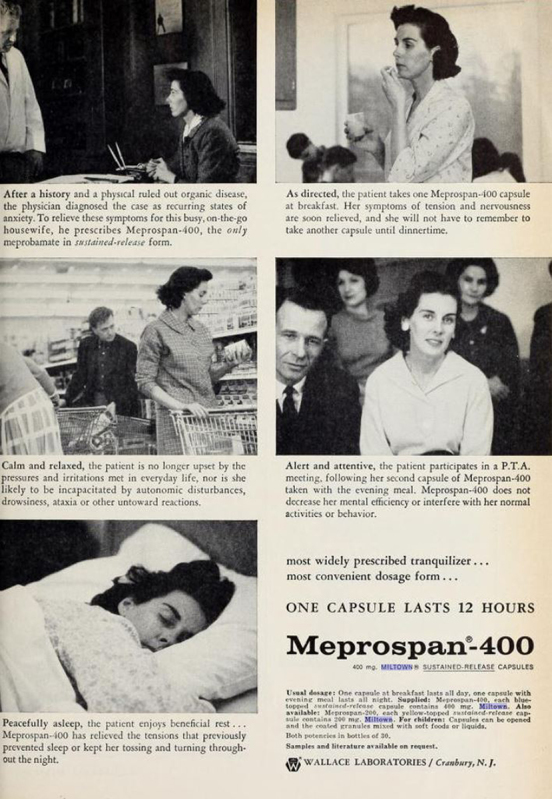
Advertisement for the drug Mesopran from the advertising section of the March 1961 issue of *California Medicine*, the journal of the California Medical Association.

The primary accomplishment of “Medicine at Ground Level” was the collaborative digitization of nearly every state medical journal in the United States, from their initiation almost to the present day. Given both the desire for the MHL to partner with the state medical societies (e.g., in promoting this rich resource to their constituent audiences) and the legal need to obtain permission to digitize in-copyright materials, this effort initially entailed multiple discussions with representatives (usually executive vice presidents or heads of publication, communication, or legal divisions, depending on the size of the society), prior to the receipt of written approval for the digitization. For this, the MHL owes a great debt to Principal Investigator Scott H. Podolsky, professor of global health and social medicine, Harvard Medical School, and director of the Center for the History of Medicine, Countway Library, who negotiated agreements on the MHL’s behalf with every medical society in the United States, with the exception of Massachusetts (which has a fully digitized and monetized *New England Journal of Medicine [NEJM]*) and New Hampshire (which never had a medical journal apart from its onetime sponsorship of *NEJM*).

The MHL was also able to include the Medical Society of the District of Columbia and the Puerto Rico Medical Association. The latter reached out after one of its members attended the MHL’s 2016 American Association for the History of Medicine (AAHM) panel, “Medicine at Ground Level from the Medical Heritage Library: State Medical Societies, State Medical Journals, and the Development of American Medicine and Society,” which had been chaired by Podolsky. Agreements with state medical societies utilized Creative Commons Attribution-NonCommercial 4.0 International (CC BY-NC 4.0) language, ensuring that users can copy, redistribute, remix, transform, and build upon MHL content for noncommercial use so long as credit is provided.

**Figure:**
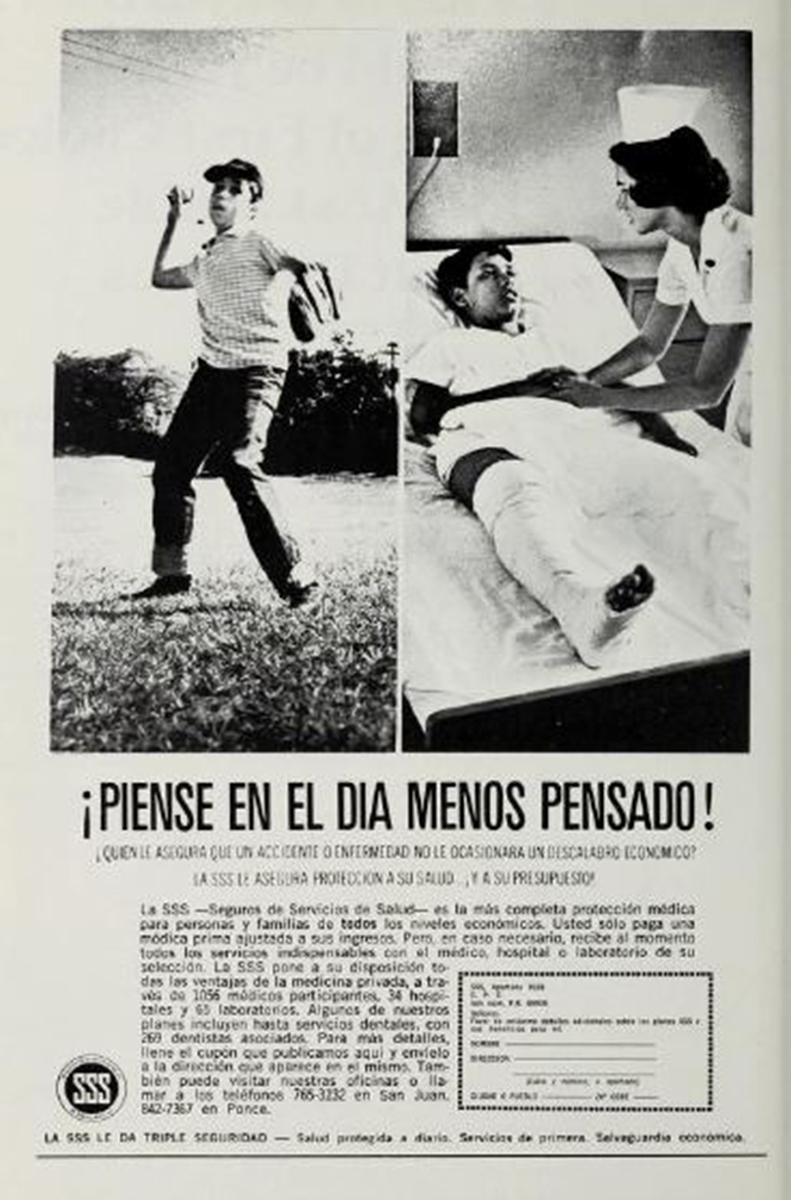
Advertisement for health insurance, from the November 1967 issue of the Boletín de la Asociación Médica de Puerto Rico, the journal of the Asociación Médica de Puerto Rico.

**Figure:**
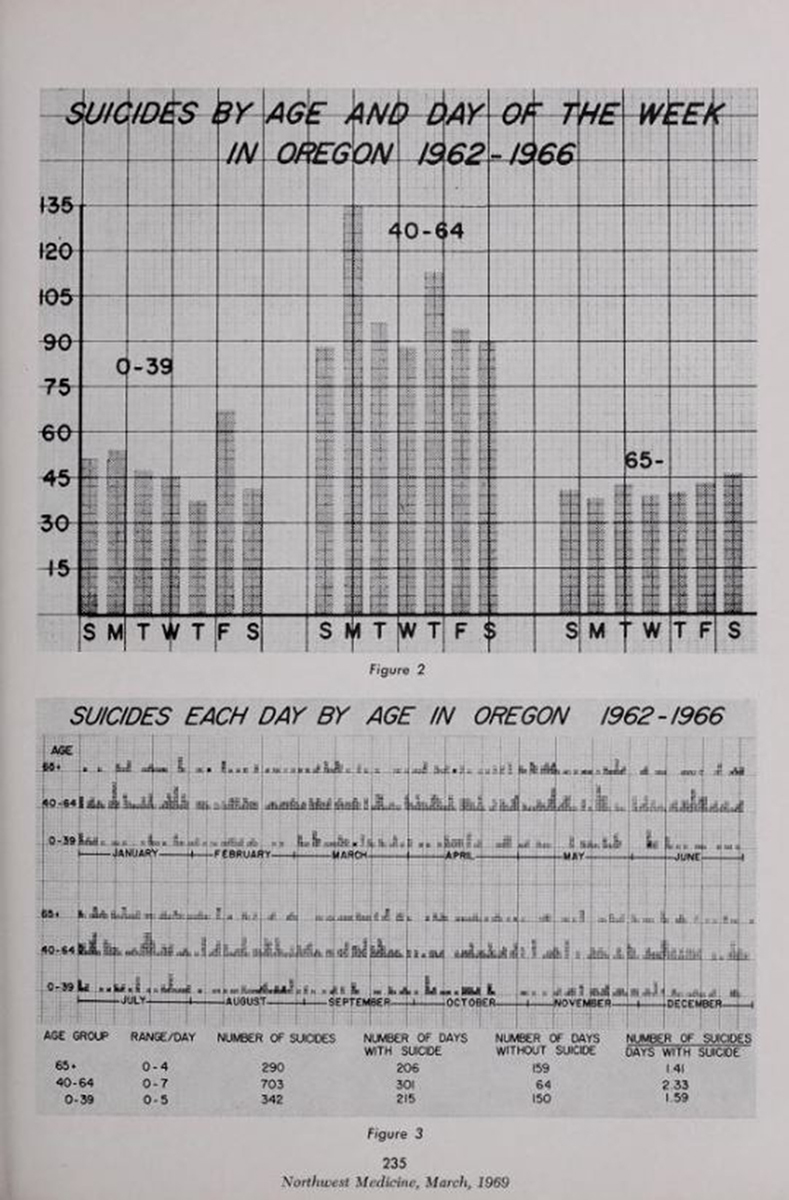
Figure from the article, “A Market Analysis for Suicide Prevention; Relationship of Age to Suicide on Holidays, Day of the Week and Month,” from the March 1969 issue of *Northwest Medicine*.

Ultimately, the five MHL partners in the project—the Center for the History of Medicine in the Francis A. Countway Library at Harvard University, the New York Academy of Medicine, the College of Physicians of Philadelphia, University of California–San Francisco, and the University of Maryland–Baltimore—digitized 97 titles from 48 states along with the District of Columbia and Puerto Rico, constituting 2,766,898 pages in 3,816 volumes, with gaps in runs continuing to be digitized and deposited to the Internet Archive through the generous efforts of the National Library of Medicine. In total, 4,666 state medical society journals are currently tagged in the Internet Archive. Beyond the Internet Archive’s access portal through which MHL content is delivered, “Medicine at Ground Level” motivated the MHL (with the input of our advisory board) to further develop the MHL’s own advanced search interface, which currently makes 260,000 items full-text searchable, complete with proximity search, date search, snippet views, and faceting by contributor, collection, and languages.

## CONCLUSION

“Medicine at Ground Level” extended the reach of the MHL and strengthened the collaborative partnership that is the MHL itself. The grant demonstrated the utility—and the labor-intensive challenges—of digitizing in-copyright items. It required investigating the legal owners of such items and demonstrating the value of making them freely available.

The MHL hopes to digitize widely read runs of “throwaway” journals (which will entail further copyright discussions), again making them full-text searchable, as well as grey literature and ephemeral printed materials that cover health issues that populations continue to grapple with today.

In an era of shared collections and massive de-duplication efforts in academic libraries that are bearing extraordinary financial pressures, making accessible unique holdings is one way that medical libraries can define themselves and formulate new services. Moreover, such collections, when meaningfully integrated into academic programs and curricula, can foster conversations around issues of equity, social justice, and diversity. We welcome the efforts of all who share our mission.

